# Germline *HOXB13* mutations p.G84E and p.R217C do not confer an increased breast cancer risk

**DOI:** 10.1038/s41598-020-65665-y

**Published:** 2020-06-16

**Authors:** Jingjing Liu, Wendy J. C. Prager - van der Smissen, J. Margriet Collée, Manjeet K. Bolla, Qin Wang, Kyriaki Michailidou, Joe Dennis, Thomas U. Ahearn, Kristiina Aittomäki, Christine B. Ambrosone, Irene L. Andrulis, Hoda Anton-Culver, Natalia N. Antonenkova, Volker Arndt, Norbert Arnold, Kristan J. Aronson, Annelie Augustinsson, Päivi Auvinen, Heiko Becher, Matthias W. Beckmann, Sabine Behrens, Marina Bermisheva, Leslie Bernstein, Natalia V. Bogdanova, Nadja Bogdanova-Markov, Stig E. Bojesen, Hiltrud Brauch, Hermann Brenner, Ignacio Briceno, Sara Y. Brucker, Thomas Brüning, Barbara Burwinkel, Qiuyin Cai, Hui Cai, Daniele Campa, Federico Canzian, Jose E. Castelao, Jenny Chang-Claude, Stephen J. Chanock, Ji-Yeob Choi, Melissa Christiaens, Christine L. Clarke, NBCS Collaborators, Kristine K. Sahlberg, Anne-Lise Børresen-Dale, Lars Ottestad, Rolf Kåresen, Ellen Schlichting, Marit Muri Holmen, Toril Sauer, Vilde Haakensen, Olav Engebråten, Bjørn Naume, Alexander Fosså, Cecile E. Kiserud, Kristin V. Reinertsen, Åslaug Helland, Margit Riis, Jürgen Geisler, Grethe I. Grenaker Alnæs, Fergus J. Couch, Kamila Czene, Mary B. Daly, Peter Devilee, Isabel dos-Santos-Silva, Miriam Dwek, Diana M. Eccles, A. Heather Eliassen, Peter A. Fasching, Jonine Figueroa, Henrik Flyger, Lin Fritschi, Manuela Gago-Dominguez, Susan M. Gapstur, Montserrat García-Closas, José A. García-Sáenz, Mia M. Gaudet, Graham G. Giles, Mark S. Goldberg, David E. Goldgar, Pascal Guénel, Christopher A. Haiman, Niclas Håkansson, Per Hall, Patricia A. Harrington, Steven N. Hart, Mikael Hartman, Peter Hillemanns, John L. Hopper, Ming-Feng Hou, David J. Hunter, Dezheng Huo, ABCTB Investigators, Christine Clarke, Deborah Marsh, Rodney Scott, Robert Baxter, Desmond Yip, Jane Carpenter, Alison Davis, Nirmala Pathmanathan, Peter Simpson, Dinny Graham, Mythily Sachchithananthan, Hidemi Ito, Motoki Iwasaki, Milena Jakimovska, Anna Jakubowska, Esther M. John, Rudolf Kaaks, Daehee Kang, Renske Keeman, Elza Khusnutdinova, Sung-Won Kim, Peter Kraft, Vessela N. Kristensen, Allison W. Kurian, Loic Le Marchand, Jingmei Li, Annika Lindblom, Artitaya Lophatananon, Robert N. Luben, Jan Lubiński, Arto Mannermaa, Mehdi Manoochehri, Siranoush Manoukian, Sara Margolin, Shivaani Mariapun, Keitaro Matsuo, Tabea Maurer, Dimitrios Mavroudis, Alfons Meindl, Usha Menon, Roger L. Milne, Kenneth Muir, Anna Marie Mulligan, Susan L. Neuhausen, Heli Nevanlinna, Kenneth Offit, Olufunmilayo I. Olopade, Janet E. Olson, Håkan Olsson, Nick Orr, Sue K. Park, Paolo Peterlongo, Julian Peto, Dijana Plaseska-Karanfilska, Nadege Presneau, Brigitte Rack, Rohini Rau-Murthy, Gad Rennert, Hedy S. Rennert, Valerie Rhenius, Atocha Romero, Matthias Ruebner, Emmanouil Saloustros, Rita K. Schmutzler, Andreas Schneeweiss, Christopher Scott, Mitul Shah, Chen-Yang Shen, Xiao-Ou Shu, Jacques Simard, Christof Sohn, Melissa C. Southey, John J. Spinelli, Rulla M. Tamimi, William J. Tapper, Soo H. Teo, Mary Beth Terry, Diana Torres, Thérèse Truong, Michael Untch, Celine M. Vachon, Christi J. van Asperen, Alicja Wolk, Taiki Yamaji, Wei Zheng, Argyrios Ziogas, Elad Ziv, Gabriela Torres-Mejía, Thilo Dörk, Anthony J. Swerdlow, Ute Hamann, Marjanka K. Schmidt, Alison M. Dunning, Paul D. P. Pharoah, Douglas F. Easton, Maartje J. Hooning, John W. M. Martens, Antoinette Hollestelle

**Affiliations:** 1 Department of Medical Oncology, Family Cancer Clinic, Erasmus MC Cancer Institute, Rotterdam, The Netherlands; 2 Institute of Medical and Pharmaceutical Sciences, Zhengzhou University, Zhengzhou, China; 3 Department of Clinical Genetics, Erasmus University Medical Center, Rotterdam, The Netherlands; 4 Centre for Cancer Genetic Epidemiology, Department of Public Health and Primary Care, University of Cambridge, Cambridge, UK; 5 Biostatistics Unit, The Cyprus Institute of Neurology & Genetics, Nicosia, Cyprus; 6 Cyprus School of Molecular Medicine, The Cyprus Institute of Neurology & Genetics, Nicosia, Cyprus; 7 Division of Cancer Epidemiology and Genetics, National Cancer Institute, National Institutes of Health, Department of Health and Human Services, Bethesda, MD USA; 8 Department of Clinical Genetics, Helsinki University Hospital, University of Helsinki, Helsinki, Finland; 9 Roswell Park Cancer Institute, Buffalo, NY USA; 10 Fred A. Litwin Center for Cancer Genetics, Lunenfeld-Tanenbaum Research Institute of Mount Sinai Hospital, Toronto, ON Canada; 11 Department of Molecular Genetics, University of Toronto, Toronto, ON Canada; 12 Department of Epidemiology, Genetic Epidemiology Research Institute, University of California Irvine, Irvine, CA USA; 13 N.N. Alexandrov Research Institute of Oncology and Medical Radiology, Minsk, Belarus; 14 Division of Clinical Epidemiology and Aging Research, German Cancer Research Center (DKFZ), Heidelberg, Germany; 15 Department of Gynaecology and Obstetrics, University Hospital of Schleswig-Holstein, Campus Kiel, Christian-Albrechts University Kiel, Kiel, Germany; 16 Institute of Clinical Molecular Biology, University Hospital of Schleswig-Holstein, Campus Kiel, Christian-Albrechts University Kiel, Kiel, Germany; 17 Department of Public Health Sciences, and Cancer Research Institute, Queen’s University, Kingston, ON Canada; 18 Department of Cancer Epidemiology, Clinical Sciences, Lund University, Lund, Sweden; 19 Cancer Center, Kuopio University Hospital, Kuopio, Finland; 20 Institute of Clinical Medicine, Oncology, University of Eastern Finland, Kuopio, Finland; 21 Translational Cancer Research Area, University of Eastern Finland, Kuopio, Finland; 22 Institute of Medical Biometry and Epidemiology, University Medical Center Hamburg-Eppendorf, Hamburg, Germany; 23 Institute of Biometry and Clinical Epidemiology, Charité –Universitätsmedizin Berlin, Berlin, Germany; 24 Department of Gynecology and Obstetrics, Comprehensive Cancer Center ER-EMN, University Hospital Erlangen, Friedrich-Alexander-University Erlangen-Nuremberg, Erlangen, Germany; 25 Division of Cancer Epidemiology, German Cancer Research Center (DKFZ), Heidelberg, Germany; 26 Institute of Biochemistry and Genetics, Ufa Federal Research Centre of the Russian Academy of Sciences, Ufa, Russia; 27 Department of Population Sciences, Beckman Research Institute of City of Hope, Duarte, CA USA; 28 Department of Radiation Oncology, Hannover Medical School, Hannover, Germany; 29 Gynaecology Research Unit, Hannover Medical School, Hannover, Germany; 30 Institute of Human Genetics, University of Münster, Münster, Germany; 31 Copenhagen General Population Study, Herlev and Gentofte Hospital, Copenhagen University Hospital, Herlev, Denmark; 32 Department of Clinical Biochemistry, Herlev and Gentofte Hospital, Copenhagen University Hospital, Herlev, Denmark; 33 Faculty of Health and Medical Sciences, University of Copenhagen, Copenhagen, Denmark; 34 Dr. Margarete Fischer-Bosch-Institute of Clinical Pharmacology, Stuttgart, Germany; 35 iFIT-Cluster of Excellence, University of Tübingen, Tübingen, Germany; 36 German Cancer Consortium (DKTK), German Cancer Research Center (DKFZ), Partner Site Tübingen, Tübingen, Germany; 37 Division of Preventive Oncology, German Cancer Research Center (DKFZ) and National Center for Tumor Diseases (NCT), Heidelberg, Germany; 38 Institute of Human Genetics, Pontificia Universidad Javeriana, Bogota, Colombia; 39 Medical Faculty, Universidad de La Sabana, Bogota, Colombia; 40 Department of Gynecology and Obstetrics, University of Tübingen, Tübingen, Germany; 41 Institute for Prevention and Occupational Medicine of the German Social Accident Insurance, Institute of the Ruhr University Bochum (IPA), Bochum, Germany; 42 Molecular Epidemiology Group, C080, German Cancer Research Center (DKFZ), Heidelberg, Germany; 43 Molecular Biology of Breast Cancer, University Womens Clinic Heidelberg, University of Heidelberg, Heidelberg, Germany; 44 Division of Epidemiology, Department of Medicine, Vanderbilt Epidemiology Center, Vanderbilt-Ingram Cancer Center, Vanderbilt University School of Medicine, Nashville, TN USA; 45 Department of Biology, University of Pisa, Pisa, Italy; 46 Genomic Epidemiology Group, German Cancer Research Center (DKFZ), Heidelberg, Germany; 47 Oncology and Genetics Unit, Instituto de Investigacion Sanitaria Galicia Sur (IISGS), Xerencia de Xestion Integrada de Vigo-SERGAS, Vigo, Spain; 48 Cancer Epidemiology Group, University Cancer Center Hamburg (UCCH), University Medical Center Hamburg-Eppendorf, Hamburg, Germany; 49 Department of Biomedical Sciences, Seoul National University Graduate School, Seoul, Korea; 50 Cancer Research Institute, Seoul National University, Seoul, Korea; 51 Leuven Multidisciplinary Breast Center, Department of Oncology, Leuven Cancer Institute, University Hospitals Leuven, Leuven, Belgium; 52 Westmead Institute for Medical Research, University of Sydney, Sydney, New South Wales Australia; 63 Department of Laboratory Medicine and Pathology, Mayo Clinic, Rochester, MN USA; 64 Department of Medical Epidemiology and Biostatistics, Karolinska Institutet, Stockholm, Sweden; 65 Department of Clinical Genetics, Fox Chase Cancer Center, Philadelphia, PA USA; 66 Department of Pathology, Leiden University Medical Center, Leiden, The Netherlands; 67 Department of Human Genetics, Leiden University Medical Center, Leiden, The Netherlands; 68 Department of Non-Communicable Disease Epidemiology, London School of Hygiene and Tropical Medicine, London, UK; 69 School of Life Sciences, University of Westminster, London, UK; 70 Faculty of Medicine, University of Southampton, Southampton, UK; 71 Channing Division of Network Medicine, Department of Medicine, Brigham and Women’s Hospital and Harvard Medical School, Boston, MA USA; 72 Department of Epidemiology, Harvard T.H. Chan School of Public Health, Boston, MA USA; 73 David Geffen School of Medicine, Department of Medicine Division of Hematology and Oncology, University of California at Los Angeles, Los Angeles, CA USA; 74 Usher Institute of Population Health Sciences and Informatics, The University of Edinburgh, Edinburgh, UK; 75 Cancer Research UK Edinburgh Centre, The University of Edinburgh, Edinburgh, UK; 76 Department of Breast Surgery, Herlev and Gentofte Hospital, Copenhagen University Hospital, Herlev, Denmark; 77 School of Public Health, Curtin University, Perth, Western Australia Australia; 78 Genomic Medicine Group, Galician Foundation of Genomic Medicine, Instituto de Investigación Sanitaria de Santiago de Compostela (IDIS), Complejo Hospitalario Universitario de Santiago, SERGAS, Santiago de Compostela, Spain; 79 Moores Cancer Center, University of California San Diego, La Jolla, CA USA; 80 Behavioral and Epidemiology Research Group, American Cancer Society, Atlanta, GA USA; 81 Medical Oncology Department, Hospital Clínico San Carlos, Instituto de Investigación Sanitaria San Carlos (IdISSC), Centro Investigación Biomédica en Red de Cáncer (CIBERONC), Madrid, Spain; 82 Cancer Epidemiology Division, Cancer Council Victoria, Melbourne, Victoria, Australia; 83 Centre for Epidemiology and Biostatistics, Melbourne School of Population and Global Health, The University of Melbourne, Melbourne, Victoria Australia; 84 Precision Medicine, School of Clinical Sciences at Monash Health, Monash University, Clayton, Victoria Australia; 85 Department of Medicine, McGill University, Montréal, QC Canada; 86 Division of Clinical Epidemiology, Royal Victoria Hospital, McGill University, Montréal, QC Canada; 87 Department of Dermatology, Huntsman Cancer Institute, University of Utah School of Medicine, Salt Lake City, UT USA; 88 Cancer & Environment Group, Center for Research in Epidemiology and Population Health (CESP), INSERM, University Paris-Sud, University Paris-Saclay, Villejuif, France; 89 Department of Preventive Medicine, Keck School of Medicine, University of Southern California, Los Angeles, CA USA; 90 Institute of Environmental Medicine, Karolinska Institutet, Stockholm, Sweden; 91 Department of Oncology, Södersjukhuset, Stockholm, Sweden; 92 Centre for Cancer Genetic Epidemiology, Department of Oncology, University of Cambridge, Cambridge, UK; 93 Department of Health Sciences Research, Mayo Clinic, Rochester, MN USA; 94 Saw Swee Hock School of Public Health, National University of Singapore and National University Health System, Singapore, Singapore; 95 Department of Surgery, National University Health System, Singapore, Singapore; 96 Department of Surgery, Kaohsiung Municipal Hsiao-Kang Hospital, Kaohsiung, Taiwan; 97 Program in Genetic Epidemiology and Statistical Genetics, Harvard T.H. Chan School of Public Health, Boston, MA USA; 98 Nuffield Department of Population Health, University of Oxford, Oxford, UK; 99 Center for Clinical Cancer Genetics, The University of Chicago, Chicago, IL USA; 100 Division of Cancer Epidemiology and Prevention, Aichi Cancer Center Research Institute, Nagoya, Japan; 101 Division of Cancer Epidemiology, Nagoya University Graduate School of Medicine, Nagoya, Japan; 102 Division of Epidemiology, Center for Public Health Sciences, National Cancer Center, Tokyo, Japan; 103 Research Centre for Genetic Engineering and Biotechnology ‘Georgi D. Efremov’, MASA, Skopje, Republic of North Macedonia; 104 Department of Genetics and Pathology, Pomeranian Medical University, Szczecin, Poland; 105 Independent Laboratory of Molecular Biology and Genetic Diagnostics, Pomeranian Medical University, Szczecin, Poland; 106 Department of Medicine, Division of Oncology, Stanford Cancer Institute, Stanford University School of Medicine, Stanford, CA USA; 107 Department of Preventive Medicine, Seoul National University College of Medicine, Seoul, Korea; 108 Division of Molecular Pathology, The Netherlands Cancer Institute - Antoni van Leeuwenhoek Hospital, Amsterdam, The Netherlands; 109 Saint Petersburg State University, Saint-Petersburg, Russia; 110 Department of Surgery, Daerim Saint Mary’s Hospital, Seoul, Korea; 111 Department of Medical Genetics, Oslo University Hospital and University of Oslo, Oslo, Norway; 112 Department of Health Research and Policy, Stanford University School of Medicine, Stanford, CA USA; 113 Epidemiology Program, University of Hawaii Cancer Center, Honolulu, HI USA; 114 Human Genetics Division, Genome Institute of Singapore, Singapore, Singapore; 115 Department of Molecular Medicine and Surgery, Karolinska Institutet, Stockholm, Sweden; 116 Department of Clinical Genetics, Karolinska University Hospital, Stockholm, Sweden; 117 Division of Population Health, Health Services Research and Primary Care, School of Health Sciences, Faculty of Biology, Medicine and Health, The University of Manchester, Manchester, UK; 118 Clinical Gerontology, Department of Public Health and Primary Care, University of Cambridge, Cambridge, UK; 119 Institute of Clinical Medicine, Pathology and Forensic Medicine, University of Eastern Finland, Kuopio, Finland; 120 Imaging Center, Department of Clinical Pathology, Kuopio University Hospital, Kuopio, Finland; 121 Molecular Genetics of Breast Cancer, German Cancer Research Center (DKFZ), Heidelberg, Germany; 122 Unit of Medical Genetics, Department of Medical Oncology and Hematology, Fondazione IRCCS Istituto Nazionale dei Tumori di Milano, Milan, Italy; 123 Department of Clinical Science and Education, Södersjukhuset, Karolinska Institutet, Stockholm, Sweden; 124 Cancer Research Malaysia, Subang Jaya, Selangor, Malaysia; 125 Department of Medical Oncology, University Hospital of Heraklion, Heraklion, Greece; 126 Department of Gynecology and Obstetrics, University of Munich, Campus Großhadern, Munich, Germany; 127 MRC Clinical Trials Unit at UCL, Institute of Clinical Trials & Methodology, University College London, London, UK; 128 Department of Laboratory Medicine and Pathobiology, University of Toronto, Toronto, ON Canada; 129 Laboratory Medicine Program, University Health Network, Toronto, ON Canada; 130 Department of Obstetrics and Gynecology, Helsinki University Hospital, University of Helsinki, Helsinki, Finland; 131 Clinical Genetics Research Lab, Department of Cancer Biology and Genetics, Memorial Sloan Kettering Cancer Center, New York, NY USA; 132 Clinical Genetics Service, Department of Medicine, Memorial Sloan Kettering Cancer Center, New York, NY USA; 133 The Breast Cancer Now Toby Robins Research Centre, The Institute of Cancer Research, London, UK; 134 Centre for Cancer Research and Cell Biology, Queen’s University Belfast, Belfast, Ireland UK; 135 Genome Diagnostics Program, IFOM - the FIRC Institute for Molecular Oncology, Milan, Italy; 136 Department of Gynaecology and Obstetrics, University Hospital Ulm, Ulm, Germany; 137 Clalit National Cancer Control Center, Carmel Medical Center and Technion Faculty of Medicine, Haifa, Israel; 138 Medical Oncology Department, Hospital Universitario Puerta de Hierro, Madrid, Spain; 139 Department of Gynaecology and Obstetrics, University Hospital Erlangen, Friedrich-Alexander University Erlangen-Nuremberg, Comprehensive Cancer Center Erlangen-EMN, Erlangen, Germany; 140 Department of Oncology, University Hospital of Larissa, Larissa, Greece; 141 Center for Familial Breast and Ovarian Cancer, Faculty of Medicine and University Hospital Cologne, University of Cologne, Cologne, Germany; 142 Center for Integrated Oncology (CIO), Faculty of Medicine and University Hospital Cologne, University of Cologne, Cologne, Germany; 143 Center for Molecular Medicine Cologne (CMMC), Faculty of Medicine and University Hospital Cologne, University of Cologne, Cologne, Germany; 144 National Center for Tumor Diseases, University Hospital and German Cancer Research Center, Heidelberg, Germany; 145 Institute of Biomedical Sciences, Academia Sinica, Taipei, Taiwan; 146 School of Public Health, China Medical University, Taichung, Taiwan; 147 Genomics Center, Centre Hospitalier Universitaire de Québec – Université Laval Research Center, Québec City, QC Canada; 148 Department of Clinical Pathology, The University of Melbourne, Melbourne, Victoria Australia; 149 Population Oncology, BC Cancer, Vancouver, BC Canada; 150 School of Population and Public Health, University of British Columbia, Vancouver, BC Canada; 151 Breast Cancer Research Programme, Cancer Research Malaysia, Subang Jaya, Selangor, Malaysia; 152 Department of Surgery, Faculty of Medicine, University of Malaya, Kuala Lumpur, Malaysia; 153 Department of Epidemiology, Mailman School of Public Health, Columbia University, New York, NY USA; 154 Department of Gynecology and Obstetrics, Helios Clinics Berlin-Buch, Berlin, Germany; 155 Department of Health Science Research, Division of Epidemiology, Mayo Clinic, Rochester, MN USA; 156 Department of Clinical Genetics, Leiden University Medical Center, Leiden, The Netherlands; 157 Department of Surgical Sciences, Uppsala University, Uppsala, Sweden; 158 Department of Medicine, Institute for Human Genetics, UCSF Helen Diller Family Comprehensive Cancer Center, University of California San Francisco, San Francisco, CA USA; 159 Center for Population Health Research, National Institute of Public Health, Cuernavaca, Morelos Mexico; 160 Division of Genetics and Epidemiology, The Institute of Cancer Research, London, UK; 161 Division of Breast Cancer Research, The Institute of Cancer Research, London, UK; 162 Division of Psychosocial Research and Epidemiology, The Netherlands Cancer Institute - Antoni van Leeuwenhoek hospital, Amsterdam, The Netherlands; 53 Department of Cancer Genetics, Institute for Cancer Research, Oslo University Hospital-Radiumhospitalet, Oslo, Norway; 54 Institute of Clinical Medicine, Faculty of Medicine, University of Oslo, Oslo, Norway; 55 Department of Research, Vestre Viken Hospital, Drammen, Norway; 56 Section for Breast- and Endocrine Surgery, Department of Cancer, Division of Surgery, Cancer and Transplantation Medicine, Oslo University Hospital-Ullevål, Oslo, Norway; 57 Department of Radiology and Nuclear Medicine, Oslo University Hospital, Oslo, Norway; 58 Department of Pathology, Akershus University Hospital, Lørenskog, Norway; 59 59Department of Tumor Biology, Institute for Cancer Research, Oslo University Hospital, Oslo, Norway; 60 Department of Oncology, Division of Surgery, Cancer and Transplantation Medicine, Oslo University Hospital-Radiumhospitalet, Oslo, Norway; 61 National Advisory Unit on Late Effects after Cancer Treatment, Oslo University Hospital-Radiumhospitalet, Oslo, Norway; 62 Department of Oncology, Akershus University Hospital, Lørenskog, Norway; 176 Department of Circulation and Medical Imaging, Norwegian University of Science and Technology (NTNU), Trondheim, Norway; 177 Department of Pathology, Oslo University Hospital, Oslo, Norway; 178 Østfold Hospital, Østfold, Norway; 179 Cancer Registry of Norway, Oslo, Oslo, Norway; 180 Akershus University College of Applied Sciences, Faculty of Health Science, Oslo, Norway; 181 Centre for Cancer Biomedicine, University of Oslo, Oslo, Norway; 182 Department of Computer Science, University of Oslo, Oslo, Norway; 183 Department of Pharmacy, Faculty of Health Sciences, University of Tromsø, Tromsø, Norway; 184 Breast and Endocrine Surgery, Department of Breast and Endocrine Surgery, Vestre Viken Hospital, Drammen, Norway; 163 Centre for Cancer Research, The Westmead Institute for Medical Research, The University of Sydney, Sydney, New South Wales Australia; 164 Translational Oncology Group, School of Life Sciences, Faculty of Science, University of Technology Sydney, Sydney, New South Wales Australia; 165 School of Biomedical Sciences, University of Newcastle, Newcastle, UK; 166 Hunter Medical Research Institute and NSW Health Pathology North, Newcastle, Australia; 167 Kolling Institute of Medical Research, University of Sydney, Sydney, New South Wales Australia; 168 Epigenetics and Transcription Laboratory, Melanie Swan Memorial Translational Centre, Sci-Tech, University of Canberra, Canberra, Australian Capital Territory, Australia; 169 Department of Medical Oncology, The Canberra Hospital, Canberra, Australia; 170 Scientific Platforms, The Westmead Institute for Medical Research, University of Sydney, Sydney, New South Wales Australia; 171 The Canberra Hospital, Canberra, Australia; 172 The Australian National University, Canberra, Australia; 173 Westmead Breast Cancer Institute, Western Sydney Local Health District, Sydney, New South Wales Australia; 174 University of Sydney, Western Clinical School, Sydney, New South Wales Australia; 175 UQ Centre for Clinical Research, Faculty of Medicine, The University of Queensland, Brisbane, Australia

**Keywords:** Cancer, Genetics, Oncology, Risk factors

## Abstract

In breast cancer, high levels of homeobox protein Hox-B13 (HOXB13) have been associated with disease progression of ER-positive breast cancer patients and resistance to tamoxifen treatment. Since *HOXB13* p.G84E is a prostate cancer risk allele, we evaluated the association between *HOXB13* germline mutations and breast cancer risk in a previous study consisting of 3,270 familial non-*BRCA1/2* breast cancer cases and 2,327 controls from the Netherlands. Although both recurrent *HOXB13* mutations p.G84E and p.R217C were not associated with breast cancer risk, the risk estimation for p.R217C was not very precise. To provide more conclusive evidence regarding the role of HOXB13 in breast cancer susceptibility, we here evaluated the association between *HOXB13* mutations and increased breast cancer risk within 81 studies of the international Breast Cancer Association Consortium containing 68,521 invasive breast cancer patients and 54,865 controls. Both *HOXB13* p.G84E and p.R217C did not associate with the development of breast cancer in European women, neither in the overall analysis (OR = 1.035, 95% CI = 0.859–1.246, *P* = 0.718 and OR = 0.798, 95% CI = 0.482–1.322, *P* = 0.381 respectively), nor in specific high-risk subgroups or breast cancer subtypes. Thus, although involved in breast cancer progression, *HOXB13* is not a material breast cancer susceptibility gene.

## Introduction

Breast cancer is a complex disease and several classes of germline variants have been identified that together explain about half of the total genetic heritability of breast cancer. These include rare germline mutations in high and moderate penetrance breast cancer susceptibility genes *BRCA1*, *BRCA2*, *CDH1*, *PTEN*, *STK11*, *TP53*, *PALB*2, *ATM*, *CHEK2* and *NBN*^1^. In addition, genome-wide association studies (GWASs) have identified over 170 common low penetrance alleles each conferring a small increased risk to develop breast cancer^2,3^. Importantly, the risks these low penetrance alleles confer combine multiplicatively and since these variants are so common in the population women in the top 1% of risk have a 4.4- and 2.8-fold increased risk to develop ER-positive and ER-negative breast cancer, respectively^4^. Still, to identify better those women at risk for developing breast cancer and establish more precise risk estimates, we need to explain the remainder of the genetic heritability of breast cancer.

In this respect, the rare *HOXB13* p.G84E germline mutation (*i.e*. NM_006361.6:c.251 G > A; NP_006352.2:p.(G84E); rs138213197:C > T) was found to be associated with an increased risk to develop prostate cancer by linkage analysis and candidate gene sequencing of 200 genes at the 17q21-22 linkage region^5^. Since then, several studies have validated this association and meta-analyses have shown the prostate cancer risk to be 3- to 4-fold increased for male carriers^6–8^. Moreover, the p.G84E mutation also associated with early-onset prostate cancer, multiple affected relatives and highly aggressive disease^6,8^. Considering the evidence, there is a strong consensus for including the *HOXB13* gene in genetic testing for hereditary prostate cancer^9^.

In recent years, we have also begun to understand the role of HOXB13 in prostate cancer progression. HOXB13 acts as a transcription factor and, together with the androgen receptor (AR) and FOXA1, regulates expression of the *RFX6* gene which encodes a driver of prostate cancer progression. Interestingly, HOXB13 is preferentially recruited to the risk allele of a prostate cancer risk associated SNP, rs339331, located in an enhancer element upstream of *RFX6*, thereby enhancing RFX6 expression and promoting more aggressive disease^10^. Moreover, HOXB13 also pioneers binding of the constitutively active splice variant 7 of the androgen receptor (AR-V7) to open chromatin of castrate-resistant prostate cancer (CRPC) genomes to upregulate target oncogenes^11^. Importantly, AR-V7 plays an important role in the anti-AR therapy resistance^12^.

In breast cancer, HOXB13 also plays an important role in disease progression. A high *HOXB13* to *IL17BR* expression ratio was associated with a high risk of recurrence and poor outcome for estrogen receptor (ER)-positive breast cancer patients^13–15^. Furthermore, high expression of HOXB13 predicted a poor response to tamoxifen therapy by suppressing ER and activating the mTOR pathway via IL6^16,17^. Interestingly, a significant fraction of breast cancer risk SNPs have been found to alter the affinity of chromatin for pioneer factor FOXA1 with which HOXB13 interacts in prostate cancer cells^10,18^. To date, several studies have investigated the association between the germline *HOXB13* p.G84E mutation and breast cancer risk, however, these have led to contradictory results^7,19–21^.

In a previous study, we have sequenced the entire coding region of *HOXB13* in 1,250 familial non-*BRCA1/2* breast cancer cases and 800 controls. We identified two recurrent *HOXB13* mutations in the female Dutch population, the known prostate cancer risk allele p.G84E, but also p.R217C (*i.e*. NM_006361.6:c.649 C > T; NP_006352.2:p.(R217C); rs139475791:G > A). We found that neither p.G84E nor p.R217C were associated with an increased breast cancer risk (OR = 0.81, 95% CI = 0.41–1.59, *P* = 0.54 and OR = 3.57, 95% CI = 0.76–33.57, *P* = 0.14, respectively) in 3,270 familial non-*BRCA1/2* breast cancer patients and 2,327 controls^22^. Considering the low carrier allele frequency (CAF; 0.09% in controls) and the very wide confidence intervals for the association between p.R217C and breast cancer risk, larger studies are needed to provide more conclusive evidence. Furthermore, we wanted to replicate our findings for the p.G84E mutation. Therefore, we have genotyped 68,521 breast cancer cases and 54,865 controls from 81 studies in the Breast Cancer Association Consortium (BCAC) for the *HOXB13* p.G84E and p.R217C mutations.

## Results

The CAF for the p.G84E mutation varies among different populations. In Asian and African BCAC studies, the p.G84E mutation was not detected, while the CAF was highest in Northern European countries (*i.e*. Sweden, Denmark and the Netherlands in controls) (Supplementary Table S1). Therefore, we restricted our analysis for the p.G84E mutation to 54,731 cases and 44,298 controls from European countries with a CAF that was larger than zero. In the overall analysis, the p.G84E mutation was not associated with breast cancer risk in Europeans (OR = 1.033, 95% CI = 0.857–1.244, *P* = 0.734; Table 1) in agreement with our previous study. We also performed analyses in which we enriched for high-risk subgroups such as women who were diagnosed before 50 years of age, premenopausal women and women with a family history of breast cancer or contralateral breast cancer. We also performed analyses by receptor status to evaluate whether *HOXB13* p.G84E associates with subtype-specific breast cancer risk. However, we did neither find any association between *HOXB13* p.G84E and the risk of breast cancer in any of these high-risk subgroups, nor did we find an association with subtype-specific breast cancer risk (Table 1).

**Table 1:** Association of *HOXB13* p.G84E with breast cancer risk in women of European descent.

	N Controls	N Cases	CAF (%) Controls	CAF (%) Cases	OR (95% CI)*	*P*-value*
**Overall analysis**						
Europeans	44,298	54,731	0.510	0.471	1.03 (0.86–1.24)	0.74
**Subgroup analysis**						
Age of diagnosis						
< 50 years	44,298	17,641	0.510	0.431	0.99 (0.72–1.35)	0.98
Menopausal status						
Premenopausal	44,298	12,134	0.510	0.503	1.20 (0.88–1.64)	0.24
Family history						
1^st^ degree relative with BC	41,876	7,582	0.533	0.462	1.04 (0.72–1.51)	0.83
Second BC						
Contralateral BC	38,310	2,144	0.506	0.373	1.00 (0.48–2.10)	0.99
Receptor status						
ER positive	44,298	35,969	0.510	0.442	0.98 (0.80–1.21)	0.88
ER negative	44,298	9,343	0.510	0.503	1.21 (0.87–1.68)	0.26
Triple negative	44,298	4,017	0.510	0.448	1.26 (0.76–2.06)	0.37

N, number; CAF, carrier allele frequency; OR, odds ratio; CI, confidence interval; BC, breast cancer; ER, estrogen receptor.

*Dominant genetic model adjusted for country, age and principal components. Not all BCAC studies had info on all variables.

Although in our previous study we found that the *HOXB13* p.R217C mutation was 3.5-fold more prevalent in cases than controls, the association between p.R217C and breast cancer risk was not statistically significant and the estimation of the risk was not very precise. Therefore, we evaluated the association of p.R217C with breast cancer risk in the 81 BCAC studies. Similar to p.G84E, the CAF for p.R217C varied among different populations. It was absent in both cases and controls of Asian ancestry, but not those of European and African ancestry. The CAF was highest in Macedonia, the Netherlands and Greece in controls (Supplementary Table S1). We analyzed 54,752 breast cancer patients and 44,422 controls from European countries with a CAF that was larger than zero. In the overall analysis, p.R217C was not associated with an increased breast cancer risk in European women (OR = 0.798, 95% CI = 0.482–1.322, *P* = 0.381; Table 2). Likewise, high-risk subgroup analyses and analyses by receptor status also did not reveal any association between *HOXB13* p.R217C and (subtype-specific) breast cancer risk (Table 2).

**Table 2:** Association of *HOXB13* p.R217C with breast cancer risk in women of European descent.

	N Controls	N Cases	CAF (%) Controls	CAF (%) Cases	OR (95% CI) *	*P*-value*
**Overall analysis**						
Europeans	44,422	54,752	0.077	0.062	0.80 (0.48–1.32)	0.38
**Subgroup analysis**						
Age of diagnosis						
< 50 years	44,422	17,669	0.077	0.045	0.38 (0.14–1.01)	0.05
Menopausal status						
Premenopausal	44,422	12,195	0.077	0.057	0.59 (0.24–1.44)	0.25
Family history						
1^st^ degree relative with BC	41,909	7,531	0.069	0.013	0.21 (0.03–1.53)	0.12
Second BC						
Contralateral BC	38,346	2,137	0.076	0.047	0.43 (0.05–3.43)	0.43
Receptor status						
ER positive	44,422	35,930	0.077	0.061	0.81 (0.46–1.42)	0.46
ER negative	44,422	9,343	0.077	0.064	0.82 (0.33–2.03)	0.66
Triple negative	44,422	4,045	0.077	0.025	0.29 (0.04–2.19)	0.23

N, number; CAF, carrier allele frequency; OR, odds ratio; CI, confidence interval; BC, breast cancer; ER, estrogen receptor.

*Dominant genetic model adjusted for country, age and principal components. Not all BCAC studies had info on all variables.

In our previous study we had sequenced the entire coding region of *HOXB13* in 1,250 familial non-*BRCA1/*2 breast cancer patients and 800 controls and identified two other, less frequent, *HOXB13* missense mutations: p.P190L (*i.e*. NM_006361.6: c.569 C > T; NP_006352.2:p.(P190L)) and p.R268Q (*i.e*. NM_006361.6:c.803 G > A; NP_006352.2:p.(R268Q); rs748782183:C > T)^22^. These two mutations had not been investigated before due to their low frequency in the Dutch population. However, the present study enabled us to assess their frequency in a global context. The p.P190L mutation was most prevalent in the African population and absent in the Asian population (Supplementary Table S1). In the Europeans, we identified only four breast cancer patients and four controls carrying this mutation. The low population frequency in Europeans and the low sample size in Africans precluded any reliable analysis of an association with breast cancer risk. The p.R268Q mutation was absent in Asian and African BCAC studies. In Europeans, we identified only two breast cancer patients and two controls carrying this mutation, again precluding any reliable analysis of an association with breast cancer risk (Supplementary Table S1).

## Discussion

We genotyped four *HOXB13* missense mutations: p.G84E, p.P190L, p.R217C and p.R268Q in 68,521 breast cancer cases and 54,865 controls from 81 studies in the BCAC on the OncoArray. All mutations were present in Europeans, but not in Asians. The p.P190L and p.R217C mutations were also present in the African ancestry BCAC studies, but not p.G84E and p.R268Q. Both p.P190L and p.R268Q were too rare to be evaluated for their association with an increased breast cancer risk. There were sufficient carriers of *HOXB13* p.G84E and p.R217C to allow association analysis in Europeans, however, both mutations did not associate with breast cancer risk. Our study, by contrast with prostate cancer, shows that *HOXB13* is not a material breast cancer susceptibility gene.

The current study is by far the largest study that has been performed evaluating the association with an increased breast cancer risk for germline *HOXB13* mutation carriers. Previously, Alanee *et al*. had found evidence that *HOXB13* p.G84E conferred an increased breast cancer risk in 877 familial non-*BRCA1/2* mutation carriers and 1650 controls (OR = 5.7, 95% CI = 1.0–40.7, *P* = 0.02)^19^. However, in a larger study conducted by Akbari *et al*., no such association between the p.G84E mutation and an increased breast cancer risk was observed among 4,037 cases, of which 1,082 were familial, and 2,762 controls (OR = 1.2, 95% CI = 0.34–4.1, *P* = 1.0)^20^. A study by Laitinen *et al*. consisting of 986 breast cancer patients (*i.e*. 323 familial non-*BRCA1/2* carriers and 663 unselected breast cancer patients) and 1,449 controls also did not reveal an association for overall breast cancer risk and p.G84E among Finnish women^21^. Results of these three studies have been pooled in a fixed-effects meta-analysis by Cai *et al*. and did not find a significant association between *HOXB13* p.G84E and an increased breast cancer risk (OR = 1.42, 95% CI = 0.78–2.61, *P* = 0.26)^7^. We also did not observe an increased breast cancer risk associated with the p.G84E mutation in our previous study of 3,270 familial non-*BRCA1/2* breast cancer cases and 2,327 controls (OR = 0.81, 95% CI = 0.41–1.59, *P* = 0.54)^22^. The results of the current study concur with these observations in that *HOXB13* p.G84E does not appear to act as a breast cancer susceptibility allele, neither in overall analyses (OR = 1.035, 95% CI = 0.859–1.246, *P* = 0.718) nor in analyses enriching for particular (high-risk) subgroups.

Besides p.G84E, we also identified p.R217C to be a recurrent mutation in the female Dutch population^22^. Since the estimation of the breast cancer risk for this mutation was not very precise in our previous study, we sought to re-evaluate the association between p.R217C and increased breast cancer risk in the current study. As for p.G84E, we did not find any association between p.R217C and an increased breast cancer risk, neither in overall analyses (OR = 0.798, 95% CI = 0.482–1.322, *P* = 0.381), nor in subgroup analyses. Interestingly, the p.R217C mutation had been described before among a few prostate cancer cases, but Xu *et al*. reported that p.R217C did not co-segregate with prostate cancer in the two families they identified^23,24^. In concordance with this, OncoArray summary association results from the PRACTICAL consortium show that, indeed, p.R217C is also not a material prostate cancer susceptibility allele (OR = 1.32, 95% CI = 0.57–2.07), while p.G84E is associated with an increased prostate cancer risk in this data set (OR = 4.23, 95% CI = 4.03–4.42)^25^.

Although HOXB13 plays an important role in both breast and prostate cancer progression^10,11,13–17^, germline mutations in the *HOXB13* gene seem to associate with the development of prostate cancer only^5–8^. This suggests distinct biological pathways associated with HOXB13 function in breast and prostate tissue. In prostate cancer, HOXB13 co-localizes with AR and acts as a repressor of AR target genes to modulate AR hormonal responses^26,27^. In breast cancer, ER and HOXB13 have been shown to regulate each other’s expression^17,28^. Thus, in both tissue types hormonal responses are closely interlinked with HOXB13 function. More research is needed, however, to understand better the differential roles of HOXB13 in disease initiation and progression.

To conclude, in our large study consisting of 68,521 invasive breast cancer cases and 54,865 controls from 81 BCAC studies we provide strong evidence that the rare, but recurrent *HOXB13* germline mutations p.G84E and p.R217C are not associated with an increased risk to develop breast cancer. *HOXB13* is therefore not a material breast cancer susceptibility gene.

## Materials and Methods

### Study population

In this study, BCAC consists of 81 case-control studies of unrelated women with participants of European, Asian and African ancestry contributing 68,521 patients with invasive breast cancer and 54,865 controls^2,3^. All studies provided core data on disease status and age at diagnosis while only a subset of the studies provided data on menopausal status, ER, PR and ERBB2 status, family history and bilateral breast cancer. All 81 BCAC studies were approved by their relevant governing research ethics committee and all participants provided written informed consent. The experimental protocol was approved by the Medical Ethical Committee of the Erasmus Medical Center Rotterdam and the study was carried out in accordance with the Code of Conduct of the Federation of Medical Scientific Societies in the Netherlands (https://www.federa.org/gedragscodes).

### OncoArray genotyping

Genotyping of the 81 BCAC studies was performed previously using the OncoArray, a custom-designed Illumina Infinium BeadChip. About half of the approximately 533,000 OncoArray SNPs were selected as a ‘GWAS backbone’ (Illumina HumanCore) with the remainder of SNPs selected by the disease-based consortia representing the main cancer sites (*e.g*. breast, ovarian, prostate, lung, colorectal) for several distinct reasons as detailed in^29^. Approximately 72,000 SNPs were selected specifically for their relevance to breast cancer. Details of the genotype calling and quality control for OncoArray are described elsewhere^2,29^. In brief, samples were excluded when the call rate was below 95% or when these were probable duplicates, close relatives or samples with extreme heterozygosity. Ancestry was computed using a principal component analysis (PCA). Variants were excluded using the following criteria: an overall call rate <99% or <95% in any consortium, minor allele frequency (MAF) < 0.001, poor intensity and clustering metrics, deviation from the expected frequency as observed in the 1000 Genomes Project and deviation from the Hardy-Weinberg equilibrium (HWE; *P* < 10^-7^ in controls or *P* < 10^-12^ in cases). A total of 494,763 SNPs passed the quality control and included the following four *HOXB13* missense variants: c.251 G > A (p.G84E; rs138213197), c.569 C > T (p.P190L), c.649 C > T (p.R217C; rs139475791) and c.803 G > A (p.R268Q).

### Statistical analyses

The association between *HOXB13* mutations and invasive breast cancer risk was evaluated using dominant genetic models by logistic regression analysis adjusting for country, age and principal components in European women. Subgroup analyses for the p.G84E and p.R217C variants were based on enriching for high-risk subgroups (*i.e* women diagnosed with breast cancer <50 years, premenopausal women, women with a family history of breast cancer (*i.e*. 1^st^ degree relative with breast cancer) and women diagnosed with a contralateral breast cancer) as well as stratification for hormone receptor status (*i.e*. ER positive, ER negative, triple negative) to evaluate subtype-specific breast cancer risk. All *P*-values were two-sided and *P* < 0.05 was considered to be statistically significant after correction for multiple testing by the Bonferroni procedure. Logistic regression analyses were performed using R version 3.3.3.

## Supplementary information

Supplementary Information.

## Data Availability

OncoArray summary statistics from the BCAC are available at http://bcac.ccge.medschl.cam.ac.uk/bcacdata/oncoarray/gwas-icogs-and-oncoarray-summary-results/. Per-sample genotype data, core demographic data and data on diagnosis and pathology can be requested via the BCAC Data Access Co-ordinating Committee (DACC) at http://bcac.ccge.medschl.cam.ac.uk/bcacdata/. OncoArray summary statistics from the PRACTICAL consortium are available at http://practical.icr.ac.uk/blog/?page_id=8088.
