# Supplementary material for: Germline HOXB13 mutations p.G84E and p.R217C do not confer an increased breast cancer risk
Source: Sci Rep. 2020 Jun 16;10:9688. doi: 10.1038/s41598-020-65665-y (PMC7297796; doi:10.1038/s41598-020-65665-y)
Supplement: Supplementary file 1 — Supplementary Information. [file 41598_2020_65665_MOESM1_ESM.docx]

**Supplementary Information**

**Germline *HOXB13* mutations p.G84E and p.R217C do not confer an increased breast cancer risk**

Jingjing Liu, Wendy J.C. Prager - van der Smissen, J. Margriet Collée, Manjeet K. Bolla, Qin Wang, Kyriaki Michailidou, Joe Dennis, Thomas U. Ahearn, Kristiina Aittomäki, Christine B. Ambrosone, Irene L. Andrulis, Hoda Anton-Culver, Natalia N. Antonenkova, Volker Arndt, Norbert Arnold, Kristan J. Aronson, Annelie Augustinsson, Päivi Auvinen, Heiko Becher, Matthias W. Beckmann, Sabine Behrens, Marina Bermisheva, Leslie Bernstein, Natalia V. Bogdanova, Nadja Bogdanova-Markov, Stig E. Bojesen, Hiltrud Brauch, Hermann Brenner, Ignacio Briceno, Sara Y. Brucker, Thomas Brüning, Barbara Burwinkel, Qiuyin Cai, Hui Cai, Daniele Campa, Federico Canzian, Jose E. Castelao, Jenny Chang-Claude, Stephen J. Chanock, Ji-Yeob Choi, Melissa Christiaens, Christine L. Clarke, NBCS Collaborators, Fergus J. Couch, Kamila Czene, Mary B. Daly, Peter Devilee, Isabel dos-Santos-Silva, Miriam Dwek, Diana M. Eccles, A. Heather Eliassen, Peter A. Fasching, Jonine Figueroa, Henrik Flyger, Lin Fritschi, Manuela Gago-Dominguez, Susan M. Gapstur, Montserrat García-Closas, José A. García-Sáenz, Mia M. Gaudet, Graham G. Giles, Mark S. Goldberg, David E. Goldgar, Pascal Guénel, Christopher A. Haiman, Niclas Håkansson, Per Hall, Patricia A. Harrington, Steven N. Hart, Mikael Hartman, Peter Hillemanns, John L. Hopper, Ming-Feng Hou, David J. Hunter, Dezheng Huo, ABCTB Investigators, Hidemi Ito, Motoki Iwasaki, Milena Jakimovska, Anna Jakubowska, Esther M. John, Rudolf Kaaks, Daehee Kang, Renske Keeman, Elza Khusnutdinova, Sung-Won Kim, Peter Kraft, Vessela N. Kristensen, Allison W. Kurian, Loic Le Marchand, Jingmei Li, Annika Lindblom, Artitaya Lophatananon, Robert N. Luben, Jan Lubiński, Arto Mannermaa, Mehdi Manoochehri, Siranoush Manoukian, Sara Margolin, Shivaani Mariapun, Keitaro Matsuo, Tabea Maurer, Dimitrios Mavroudis, Alfons Meindl, Usha Menon, Roger L. Milne, Kenneth Muir, Anna Marie Mulligan, Susan L. Neuhausen, Heli Nevanlinna, Kenneth Offit, Olufunmilayo I. Olopade, Janet E. Olson, Håkan Olsson, Nick Orr, Sue K. Park, Paolo Peterlongo, Julian Peto, Dijana Plaseska-Karanfilska, Nadege Presneau, Brigitte Rack, Rohini Rau-Murthy, Gad Rennert, Hedy S. Rennert, Valerie Rhenius, Atocha Romero, Matthias Ruebner, Emmanouil Saloustros, Rita K. Schmutzler, Andreas Schneeweiss, Christopher Scott, Mitul Shah, Chen-Yang Shen, Xiao-Ou Shu, Jacques Simard, Christof Sohn, Melissa C. Southey, John J. Spinelli, Rulla M. Tamimi, William J. Tapper, Soo H. Teo, Mary Beth Terry, Diana Torres, Thérèse Truong, Michael Untch, Celine M. Vachon, Christi J. van Asperen, Alicja Wolk, Taiki Yamaji, Wei Zheng, Argyrios Ziogas, Elad Ziv, Gabriela Torres-Mejía, Thilo Dörk, Anthony J. Swerdlow, Ute Hamann, Marjanka K. Schmidt, Alison M. Dunning, Paul D.P. Pharoah, Douglas F. Easton, Maartje J. Hooning, John W.M. Martens, Antoinette Hollestelle

**Supplementary Table S1.** The carrier allele frequency of *HOXB13* mutations per country from 81 BCAC studies.

| **Ethnicity** | **Country** | **CAF () p.G84E** | | **CAF () p.P190L** | | **CAF () p.R217C** | | **CAF () p.R268Q** | |
| --- | --- | --- | --- | --- | --- | --- | --- | --- | --- |
|  |  | **Controls** | **Cases** | **Controls** | **Cases** | **Controls** | **Cases** | **Controls** | **Cases** |
| **European** |  |  |  |  |  |  |  |  |  |
|  | Australia | 0.295 | 0.188 | 0.042 | 0.027 | 0.084 | 0.027 | 0 | 0 |
|  | Belarus | 0 | 0 | 0 | 0 | 0 | 0 | 0 | 0 |
|  | Belgium | 0.079 | 0.638 | 0 | 0 | 0.158 | 0.128 | 0 | 0 |
|  | Canada | 0.327 | 0.283 | 0 | 0 | 0.065 | 0.107 | 0 | 0 |
|  | Denmark | 0.838 | 0.710 | 0 | 0 | 0.140 | 0 | 0 | 0 |
|  | Finland | 0.474 | 1.368 | 0 | 0 | 0 | 0 | 0 | 0 |
|  | France | 0.189 | 0.152 | 0 | 0 | 0.189 | 0 | 0 | 0 |
|  | Germany | 0.291 | 0.495 | 0 | 0.017 | 0.112 | 0.068 | 0 | 0 |
|  | Greece | 0 | 0 | 0 | 0 | 0.195 | 0 | 0.195 | 0 |
|  | Israël | 0 | 0 | 0 | 0 | 0 | 0 | 0 | 0 |
|  | Italy | 0.173 | 0 | 0 | 0 | 0.173 | 0 | 0 | 0 |
|  | Macedonia | 0 | 0 | 0 | 0 | 1.087 | 0 | 0 | 0 |
|  | Netherlands | 0.738 | 0.573 | 0 | 0.088 | 0.285 | 0.309 | 0 | 0.044 |
|  | Norway | NA | 0.576 | NA | 0 | NA | 0.164 | NA | 0 |
|  | Poland | 0.180 | 0.048 | 0 | 0 | 0 | 0.048 | 0 | 0 |
|  | Russia | 0 | 0 | 0 | 0 | 0 | 0 | 0 | 0 |
|  | Spain | 0.097 | 0 | 0 | 0 | 0.097 | 0 | 0 | 0 |
|  | Sweden | 0.959 | 1.047 | 0.024 | 0 | 0.012 | 0.019 | 0 | 0 |
|  | UK | 0.568 | 0.458 | 0.018 | 0 | 0.055 | 0.131 | 0 | 0 |
|  | USA | 0.446 | 0.403 | 0 | 0 | 0.059 | 0.029 | 0.007 | 0.007 |
| **Asian** |  |  |  |  |  |  |  |  |  |
|  | China | 0 | 0 | 0 | 0 | 0 | 0 | 0 | 0 |
|  | Hong Kong | 0 | 0 | 0 | 0 | 0 | 0 | 0 | 0 |
|  | Japan | 0 | 0 | 0 | 0 | 0 | 0 | 0 | 0 |
|  | Korea | 0 | 0 | 0 | 0 | 0 | 0 | 0 | 0 |
|  | Malaysia | 0 | 0 | 0 | 0 | 0 | 0 | 0 | 0 |
|  | Singapore | 0 | 0 | 0 | 0 | 0 | 0 | 0 | 0 |
|  | Taiwan | 0 | 0 | 0 | 0 | 0 | 0 | 0 | 0 |
|  | Thailand | 0 | 0 | 0 | 0 | 0 | 0 | 0 | 0 |
| **African** |  |  |  |  |  |  |  |  |  |
|  | Cameroon, Nigeria, Uganda | 0 | 0 | 0 | 0.317 | 0 | 0.317 | 0 | 0 |
| **Other** |  |  |  |  |  |  |  |  |  |
|  | Colombia | 0 | 0 | 0 | 0 | 0 | 0 | 0 | 0 |
|  | Mexico | 0 | 0 | 0 | 0 | 0 | 0 | 0 | 0 |

CAF, carrier allele frequency; NA, not available
